# The ubiquitin-activating enzyme, UBA1, as a novel therapeutic target for AML

**DOI:** 10.18632/oncotarget.26153

**Published:** 2018-09-28

**Authors:** Samir H. Barghout, Aaron D. Schimmer

**Affiliations:** Princess Margaret Cancer Centre, University Health Network, Toronto, ON, Canada; Department of Medical Biophysics, Faculty of Medicine, University of Toronto, Toronto, ON, Canada

**Keywords:** Ubiquitin, UBA1, TAK-243, MLN7243, AML

The clinical approval of bortezomib, the first proteasome inhibitor, marked the advent of a new generation of anticancer drugs that target broadly-acting non-oncogenic cellular machineries important for both normal and cancer cells [1, 2]. Although perhaps initially surprising, targeting the highly conserved ubiquitin-proteasome system (UPS) preferentially produced proteotoxic stress in malignant cells over normal tissue and created a therapeutic window in specific cancers.

**Figure 1:**
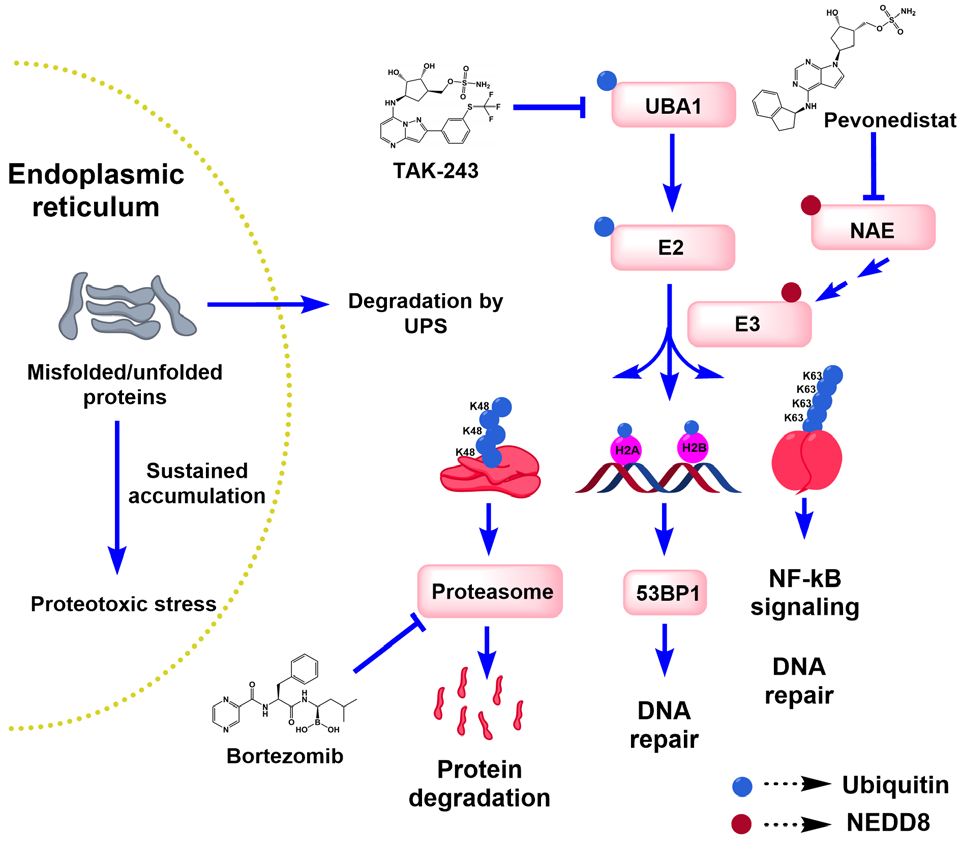
The ubiquitin-proteasome system (UPS) and its role in proteotoxic and DNA damage stress Endoplasmic reticulum-associated degradation mediated by the UPS is known to rid the cells of misfolded proteins, whose accumulation would otherwise induce proteotoxic stress, unfolded protein response and ultimately cell death after sustained disruption. Ubiquitylation is also involved in regulating DNA repair and other signaling pathways such as the NF-κB pathway. Three examples of the different ubiquitylation patterns are depicted in the diagram: mono-ubiquitin, Lys48- (K48)- and Lys63 (K63)-linked polyubiquitin chains. UBA1, NAE and the proteasome are targeted by TAK-243, pevonedistat and bortezomib, respectively.

Given the success of bortezomib, attention turned to evaluating other components of the UPS. This system consists of ubiquitin-activating enzymes (E1), ubiquitin-conjugating enzymes (E2), ubiquitin ligases (E3) and the proteasome. These three enzyme classes act sequentially to tag protein substrates with different forms of mono- or polyubiquitin. The UPS plays a key role in maintaining cellular proteostasis and regulating many ubiquitin-dependent signaling pathways such as DNA repair and nuclear factor kappa B signaling (Figure 1). We focused on targeting the initiating ubiquitin-activating enzyme, UBA1. Previously, we showed that leukemia cell lines and primary AML samples had increased reliance on UBA1 and decreased reserve capacity of the enzyme [3], suggesting inhibiting UBA1 could be of value in the treatment of AML.

Recently, we evaluated a first-in-class small-molecule UBA1 inhibitor developed by Takeda Pharmaceuticals, TAK-243, in preclinical models of AML [4]. TAK-243 is an adenosine sulphamate related to pevonedistat, a NEDD8-activating enzyme (NAE) inhibitor and the prototype of this class of mechanism-based E1 inhibitors [5]. TAK-243 acts by a unique mechanism known as substrate-assisted inhibition as it forms covalent adduct with ubiquitin that mimics the ubiquitin-adenylate complex and inhibits the cognate E1 enzyme [6].

In our study, we assessed the cytotoxicity of TAK-243 in a panel of AML cell lines and primary AML samples. TAK-243 exhibited a potent and rapid anti-leukemic activity with half-maximal inhibitory concentrations in the nanomolar range. Moreover, TAK-243 was equally effective in AML patient samples with high risk molecular and cytogenetic mutations. TAK-243 also showed preferential cytotoxicity towards leukemic versus normal hematopoietic progenitors. *In vivo*, TAK-243 reduced the leukemic burden without evidence of toxicity. To confirm the cellular target and determine the selectivity of TAK-243, we exploited the cellular thermal shift assay (CETSA) that measures target engagement in intact cells [7]. By CETSA, TAK-243 preferentially bound UBA1 over related E1 enzymes in AML cells and primary samples at concentrations associated with cell death.

We also evaluated the downstream biological effects of UBA1 inhibition after TAK-243 treatment. In AML cells and primary samples, TAK-243 reduced the abundance of ubiquitylated proteins leading to endoplasmic reticulum stress that was functionally important for TAK-243-induced cell death.

In addition, TAK-243 reduced the abundance of mono-ubiquitylated histones H2A and H2B. Mono-ubiquitylation of histones plays a pivotal role in recruitment of DNA repair molecules such as 53BP1 and BRCA1 to double-strand break (DSB) lesions [8]. As an inhibitor of histone ubiquitylation, TAK-243 inhibited DSB repair and induced DNA damage stress as evidenced by induction of γH2AX under unirradiated conditions. It also reduced 53BP1 foci at ionizing radiation-induced DSBs. Additionally, pre-treatment of AML cells with TAK-243 reduced their ability to resolve DSBs as evidenced by sustained γH2AX foci. A prior study showed that TAK-243 synergized with radiation in patient-derived breast and non-small cell lung cancer cells *in vivo* [9].

To understand mechanisms of resistance to TAK-243, we generated populations of AML cells resistant to the drug. By sequencing the adenylation domain of *UBA1* in these resistant cells, we identified Y583C and A580S missense mutations. The A580S mutation is similar to the mutation previously reported to confer resistance to pevonedistat [10]; however, the Y583C mutation is TAK-243-specific and likely conferred acquired resistance to the drug by eliminating hydrogen bonds and destabilizing the hydrophobic core of UBA1.

Recently, Hyer *et al*. characterized the mode of action and biological activity of TAK-243 in cell-free and cell-based systems and evaluated the preclinical efficacy in several mouse models of solid (prostate, ovarian, breast, colon, neck and lung) and hematologic (multiple myeloma and lymphoma) malignancies, further demonstrating efficacy and tolerability of this drug [9].

Thus, data by our group and others support advancing TAK-243 to a phase 1 clinical trial in AML patients. However, several open questions remain to be answered: 1) given its pleiotropic activity, what is the most critical mechanism of TAK-243 cytotoxicity and does it vary between different malignancies? 2) what are the determinants of sensitivity to TAK-243 in various cancers? and 3) given the anticipated broader spectrum of signaling affected by TAK-243, can this drug overcome specific forms of resistance to bortezomib and pevonedistat? Answering these questions will provide deeper insights into TAK-243 action and enable identification of potential biomarkers to determine patients most likely to benefit from this drug.
